# Unique, dual-indexed sequencing adapters with UMIs effectively eliminate index cross-talk and significantly improve sensitivity of massively parallel sequencing

**DOI:** 10.1186/s12864-017-4428-5

**Published:** 2018-01-08

**Authors:** Laura E. MacConaill, Robert T. Burns, Anwesha Nag, Haley A. Coleman, Michael K. Slevin, Kristina Giorda, Madelyn Light, Kevin Lai, Mirna Jarosz, Matthew S. McNeill, Matthew D. Ducar, Matthew Meyerson, Aaron R. Thorner

**Affiliations:** 1 Center for Cancer Genome Discovery, DFCI, 450 Brookline Avenue, Dana840b, Boston, MA 02215 USA; 2 Department of Pathology, Brigham and Women’s Hospital, Boston, MA USA; 3 Integrated DNA Technologies, Inc., Redwood City, CA USA; 4 Integrated DNA Technologies, Inc., Coralville, IA USA; 5 Department of Medical Oncology, Dana-Farber Cancer Institute (DFCI), Boston, MA USA; 6 Broad Institute of Harvard and MIT, Cambridge, MA USA

**Keywords:** Next generation sequencing, Massively parallel sequencing, Adapter, Index, Barcode cross-talk, Illumina, Multiplexing, UMI, Molecular barcode

## Abstract

**Background:**

Sample index cross-talk can result in false positive calls when massively parallel sequencing (MPS) is used for sensitive applications such as low-frequency somatic variant discovery, ancient DNA investigations, microbial detection in human samples, or circulating cell-free tumor DNA (ctDNA) variant detection. Therefore, the limit-of-detection of an MPS assay is directly related to the degree of index cross-talk.

**Results:**

Cross-talk rates up to 0.29% were observed when using standard, combinatorial adapters, resulting in 110,180 (0.1% cross-talk rate) or 1,121,074 (0.29% cross-talk rate) misassigned reads per lane in non-patterned and patterned Illumina flow cells, respectively. Here, we demonstrate that using unique, dual-matched indexed adapters dramatically reduces index cross-talk to ≤1 misassigned reads per flow cell lane. While the current study was performed using dual-matched indices, using unique, dual-unrelated indices would also be an effective alternative.

**Conclusions:**

For sensitive downstream analyses, the use of combinatorial indices for multiplexed hybrid capture and sequencing is inappropriate, as it results in an unacceptable number of misassigned reads. Cross-talk can be virtually eliminated using dual-matched indexed adapters. These results suggest that use of such adapters is critical to reduce false positive rates in assays that aim to identify low allele frequency events, and strongly indicate that dual-matched adapters be implemented for all sensitive MPS applications.

**Electronic supplementary material:**

The online version of this article (10.1186/s12864-017-4428-5) contains supplementary material, which is available to authorized users.

## Background

Massively parallel sequencing (MPS) has gained widespread use in both research and clinical laboratories for a variety of applications, including the identification of genomic factors contributing to tumorigenesis at the DNA level (such as somatic mutations, DNA insertions and deletions, and structural variants), the RNA level (transcriptome alterations), or epigenetically, among others [1–4]. Because of the large number of sequence reads generated on an MPS platform, particularly using Illumina technology (e.g., up to five billion single reads on a HiSeq 4000 system), samples are often pooled together for sequencing, prior to which each sample’s DNA fragments are tagged using sample-specific DNA indices, or “sample barcodes.” Indexing most often occurs during the library construction process, whereby fragmented genomic DNA (gDNA) is flanked by sequencing adapter constructs [5]. Illumina adapters typically contain either one (i7 index) or two index sequences (i7 and i5 indices) for each sample library. Prepared libraries may then be pooled (multiplexed), sequenced on the same flow cell lane, and subsequently deconvoluted (de-multiplexed) computationally, resulting in significant cost savings and experiment scalability [6].

Unfortunately, sample multiplexing suffers the inherent risk of index misassignment (cross-talk), which occurs when an index in the library pool is inappropriately matched to a sequence read derived from a different sample in the pool. Index cross-talk can be introduced by a variety of mechanisms, including the following: errors or cross-contamination introduced by the manufacturer during adapter oligonucleotide synthesis, purification, diluting, or aliquoting; experimental/sample handling issues; library PCR amplification error; “jumping PCR” (index hopping) during multiplex capture enrichment (Additional file 1: Figure S1); “spreading-of-signal” on patterned flow cells (flow cells in which cluster generation occurs in nanowells at fixed locations rather than the random cluster generation on non-patterned flow cells); sequencing error (e.g., insertions, substitutions, or deletions introduced during bridge amplification, or misread bases); improper cluster resolution (e.g., mixed clusters); carryover from previous runs on the same instrument; and bioinformatic errors [5, 7–12].

Cross-talk has been estimated to occur at frequencies from 0.06% to as high as 10% [7, 11, 13, 14]. Adapter cross-talk can result in false positive calls when MPS is used for sensitive applications such as low-frequency somatic variant discovery, ancient DNA investigations, microbial detection in human samples (e.g., presence of viral DNA in human tumor samples), or circulating cell-free tumor DNA (ctDNA) variant detection [5, 14, 15]. Therefore, the limit-of-detection of an MPS assay is in part related to the amount of index cross-talk.

There are several available approaches to reduce the likelihood of index cross-talk. Index sequences can be constructed so that they are less prone to errors during synthesis, amplification, and sequencing; for example, using Hamming codes [16] to reduce noise from nucleotide substitution, and Levenshtein distance [9] to account for substitutions, insertions, and deletions. In addition, when sequencing only a few samples (low-plex multiplexing) in a single Illumina flow cell lane, indices should be chosen so that each position of the index results in signal in both the red and green color channels to maintain sufficient color diversity and avoid “registration failure” [17]. Using dual indexed adapters [5] instead of single indexed adapters, and applying stringent quality filtering [7] lessens cross-talk. Furthermore, to reduce index hopping or spreading-of-signal, it is necessary to adequately remove all unbound adapters and adapter dimers before performing multiplex capture and sequencing, as index hopping occurs during post-capture PCR [11]. However, even if all aforementioned techniques are implemented, adapter cross-talk is still present due to cross-contamination introduced during many stages of adapter and library processing; this impedes the use of MPS assays where exquisite sensitivity is required.

**Fig. 1:**
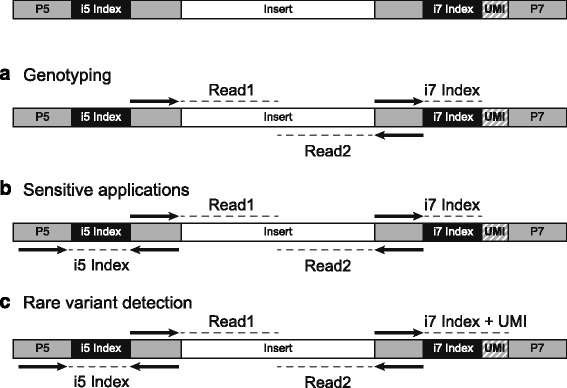
Unique dual-matched UMI adapters are compatible with shear ligation library construction methods and may be sequenced in three different modes depending on the sensitivity of the application. **a** For genotyping applications, the i7 sample index may be used for demultiplexing. **b** More sensitive applications, such as somatic variant calling, should use the i7 and i5 index. **c** When a unique molecular identifier (UMI) is required for greater sensitivity, the read length for the i7 index may be increased to sequence the sample index and UMI in addition to the i5 sample index

Recently, Integrated DNA Technologies, Inc. (IDT) made available unique, dual-matched indexed adapters with Unique Molecular Indices (UMI) designed to significantly reduce index misassignment. The adapter oligo sequence is the same as the standard Illumina TruSeq HT (“TS-96”) dual indexed adapter; however, for each individual adapter, the i5 and i7 index sequences are identical, resulting in both the forward and reverse read indices having the same sequence, and the adapter contains a UMI appended to the 3′ end of the i7 index for molecule barcoding (Fig. 1). The UMIs are short (6 nucleotide) DNA sequences that are used to tag each DNA library molecule prior to library amplification. The UMI sequences can then be used post-sequencing to aid in the identification of PCR duplicates [18]. The end user has the option to simply sequence the i7 sample index for demultiplexing in genotyping applications (Fig. 1a), both the i7 and i5 for more sensitive applications (Fig. 1b), or for greater sensitivity, the UMI can be used in addition to the i7 and i5 indices (Fig. 1c).

**Fig. 2:**
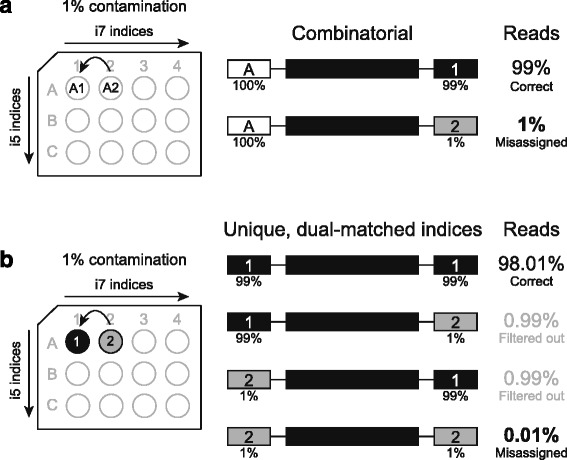
Unique dual-matched sample indices reduce read misassignment caused by contamination. **a** Example contamination of A1 adapter with 1% of A2 adapter. Because only the i7 index is used to discriminate between the samples, 1% of sample A1 reads would be misassigned to sample A2. **b** The same 1% level of contamination with unique dual-matched indexed adapters results in only 0.01% read misassignment to sample A2

Unique dual-matched indices reduce read misassignment caused by contamination because both the i5 and i7 indices on a library insert are identical but distinct from other dual-matched indexed libraries in the hybrid capture pool. Mismatched indices indicate a cross-talk error resulting in exclusion of the insert from downstream analysis. Standard, combinatorial adapters use 20 different adapters to create 96 unique combinations such that the i5 and i7 sequences are different (Additional file 2: Figure S2). If the A1 combinatorial adapter is contaminated with 1% A2 adapter, then 1% of sample A1 reads would be misassigned to A2 because only the i7 index is used to discriminate between the samples (Fig. 2a). However, if this same 1% contamination scenario occurs when using unique, matched dual-indices, the sample misidentification rate is approximately the square of the contamination level (0.01%) (Fig. 2b). Therefore, many common contamination events will be filtered out when using the dual-matched indexed adapters versus the combinatorial adapters (Additional file 3: Figure S3).

Here, we demonstrate the benefit of using unique, dual-matched adapters to significantly reduce index cross-talk, thereby vastly decreasing the MPS noise and allowing its use in sensitive applications. We determined that these adapters remove virtually all noise that may be introduced by a variety of cross-talk mechanisms, including contamination, index hopping during multiplexed hybrid capture or cluster amplification, and demultiplexing errors.

## Results

**Fig. 3:**
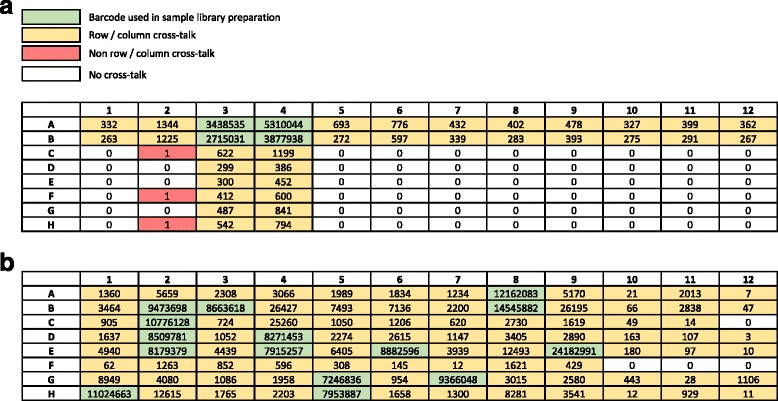
Level of cross-talk using combinatorial indices on the Illumina MiSeq and 2500 platforms. The 96-well plate layout represents the actual adapter plate arrangement. Numbers in each well represent the number of fragments that passed standard Illumina filters and demultiplexed using only perfect index matches on all TS-96 indices. **a** Four cell-line libraries were prepared using Illumina-synthesized TS-96 adapters (green), libraries were pooled, hybrid captured using a custom bait set, and then sequenced on an Illumina MiSeq v2 flow cell. **b** Fifteen patient-derived xenograft (PDX) libraries were prepared using IDT-synthesized TS-96 adapters (green), libraries were pooled, hybrid captured using a custom bait set, and then sequenced on a single lane of an Illumina HiSeq2500 flow cell

A series of experiments were conducted using either combinatorial, Illumina- or IDT-synthesized “TS-96” adapters or IDT dual-matched index adapters with UMIs to determine the amount of index cross-talk in each. First, four cell-line libraries were prepared using Illumina-synthesized TS-96 adapters, libraries were pooled, hybrid captured using a custom bait set, and then sequenced on an Illumina MiSeq v2 flow cell. Libraries passing standard Illumina filters were demultiplexed using only perfect index matches on all TS-96 indices. Next, we counted the number of fragments for each index. A total of 16,687 indices out of 15,358,235 were misassigned, for an overall index misassignment rate of 0.10% (Fig. 3a). Index cross-talk mainly occurred within the same rows and columns as the indices used to prepare the libraries. Indeed, the number of reads misassigned to unused indices residing in the same column or row as a used index ranged from 263 to 1344 (Fig. 3a). Non-row/column cross-talk was observed at a much lower rate (i.e., 0–1 misassigned indices; overall misassignment of 1.95 × 10^-5%) (Fig. 3a).

To test if index cross-talk occurred at a similar rate on the HiSeq 2500, a larger experiment was conducted. A total of 15 patient-derived xenografts (PDX) underwent library construction using randomly chosen IDT-synthesized TS-96 adapters (i.e., from a variety of adapter plate well locations), all libraries were pooled for hybrid capture, and the captured libraries were sequenced on one lane of a HiSeq 2500 in Rapid Run Mode. Again, Illumina pass filter reads were demultiplexed using only perfect index matches on all 96 indices. A total of 244,302 reads out of 157,398,602 were misassigned (Fig. 3b), for an overall quantity of index cross-talk of 0.16%. Cross-talk was observed in all but four of the 81 unused indices, and the highest levels of cross-talk tended, in general, to be directly proportional to the proximity of each index in the plate (Fig. 3b). The number of incorrectly assigned reads ranged from 0 to 26,427 per unused index (Fig. 3b). To further validate this finding, we performed a similar experiment on 19 PDX samples using adapters from the first three rows on the TS-96 plate and observed comparable results (Additional file 4: Figure S4). The total number of misassigned reads was 36,176 out of 160,295,450 total reads, and ranged from 0 to 4922 misassigned reads per index. The overall amount of cross-talk was lower (0.02%) in this experiment, which may be because we were unable to detect the cross-talk occurring in adjacent wells to the sequenced libraries, the library construction was cleaner, there was less free adapter, etc.

**Fig. 4:**
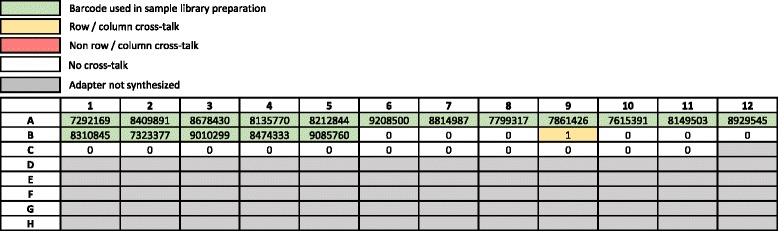
Level of cross-talk using unique, dual-matched indexed adapters on Illumina HiSeq 2500. The 96-well plate layout represents the adapter plate. A total of 35 adapters were synthesized. Seventeen human cell line libraries were prepared using IDT-synthesized unique, dual-matched indexed adapters (green), libraries were pooled, hybrid captured using a custom bait set, and then sequenced on a single lane of an Illumina HiSeq2500 flow cell. Numbers in each well represent the number of fragments that passed standard Illumina filters and demultiplexed using only perfect sequence matches on all TS-96 indices

To determine the level of index cross-talk when using dual-matched adapters, a total of 35 IDT-synthesized dual-matched indexed adapters with UMIs, which were the total number of adapters available for testing at the time, were used during the library construction of 17 human cell line samples. Because these are 35 unique indices, each adapter has a different index pair than all other adapter used (i.e., neither the i5 nor i7 indices are shared by any other adapter on the adapter plate). All 17 libraries were pooled for custom hybrid capture, and Illumina pass filter reads were demultiplexed using only perfect index matches on all 35 dual-matched indices. Remarkably, there was only a single sequenced fragment (out of a total of 141,312,388 reads) that was demultiplexed for one unused index, which is a percent contamination rate of only 7.1 × 10^-7% (Fig. 4). In addition, we searched for misassignments with all possible combinations of the 35 indices (i.e., 35 × 35 = 1225 combinations) and determined the overall rate of index cross-talk (where either i5 or i7 indices were individually mismatched) to be 0.12%, which was similar to the rate observed when using combinatorial adapters (0.16%). We performed a similar experiment using a total of 34 samples consisting of a combination of PDX (*n* = 4), tissue samples (*n* = 20), and cancer cell lines (*n* = 10). The one unused index in this experiment received zero reads (0% cross-talk; Additional file 5: Figure S5). Again, the underlying cross-talk rate was 0.12% when examining all 1225 possible index combinations.

**Fig. 5:**
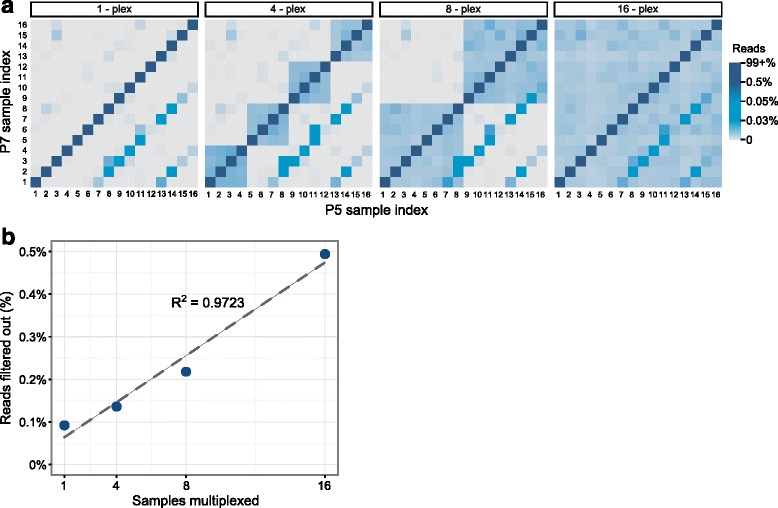
Unique dual-matched indices accurately identify contamination and index hopping events. **a** Heatmap displays the percent of reads for all i5 and i7 sample index combinations in 1-, 4-, 8- or 16-plex captures. The contamination level of library adapters was 0.09% for single library captures but increased up to 0.39% for 16-plex captures. **b** Percentage of reads filtered out per multiplex experiment when using dual-matched indexed adapters that would have been misassigned using combinatorial indices

We next sought to measure multiplex capture index hopping using unique, dual-matched adapters. A total of 16 libraries were prepared and captured using the IDT xGen AML Cancer Panel in pools of 1, 4, 8, or 16, and sequenced on separate Illumina NextSeq lanes (Additional file 6: Figure S6). The multiplexed captures had comparable uniformity to single-plex captures (Additional file 7: Figure S7). Index hopping was estimated by analyzing the percent of reads with mismatched i7 and i5 indices. The contamination level of indices was 0.09% for single-plex samples, which is likely adapter contamination, not index hopping. In the multiplexing experiments, after correcting for the adapter contamination noted above (0.09%), we observed 0.04–0.39% index hopping levels where either i5 or i7 was mismatched (Fig. 5a). However, these reads would be correctly removed from analysis when using the matched dual-index adapters (Fig. 5b). Index-hopping increased linearly with higher multiplexing, up to 0.39% for 16-plexed samples. In Fig. 5a, some index combinations were observed off-diagonal (e.g., 1, 7, and 13; or 2, 8, and 14) at higher rates, and it is important to note that those oligos were arrayed in adjacent wells during olio annealing (Additional file 8: Figure S8). This suggests that contamination was likely introduced during annealing. Indeed, if contamination occurred when the adapters were arrayed as single stranded oligonucleotides, then a skewed pattern would be expected. Alternatively, if cross-contamination occurred after duplexing, as displayed in Fig. 2, a symmetrical pattern would be expected. Hence, these results (Fig. 5a) support that cross-contamination occurred when the adapters were single stranded, prior to duplexing. However, sample read misassignment would be dramatically reduced using dual-matched indices.

A recent study posted on bioRxiv suggested that there was increased index hopping when samples were sequenced on a HiSeq 3000/4000 relative to a HiSeq 2500 [11]. To address this issue, we prepared libraries from two human tumor samples using IDT-synthesized TS-96 combinatorial adapters. The libraries were pooled, captured using a whole exome bait set, and sequenced in one lane of the HiSeq 2500 and one lane of the HiSeq 3000/4000. Illumina pass filter reads were demultiplexed using only perfect matches on all TS-96 indices. Index cross-talk was observed almost entirely a row/column pattern (Additional file 9: Figure S9). The overall amount of cross-talk increased 3-fold, and up to 10-fold more missassigned reads for a given index, for samples sequenced on the HiSeq 3000 (1,121,074 missassigned reads out of 382,464,375 total reads; 0–548,358 misassigned reads per index; 0.29% overall cross-talk) relative to the HiSeq 2500 (110,180 total misassigned reads out of 108,416,103 total reads; 0–49,533 misassigned reads per index; 0.10% overall cross-talk). While this amount of index hopping was slightly less than previously reported [11], the trend was the same, and dual-matched indices should effectively decrease this cross-talk.

**Fig. 6:**
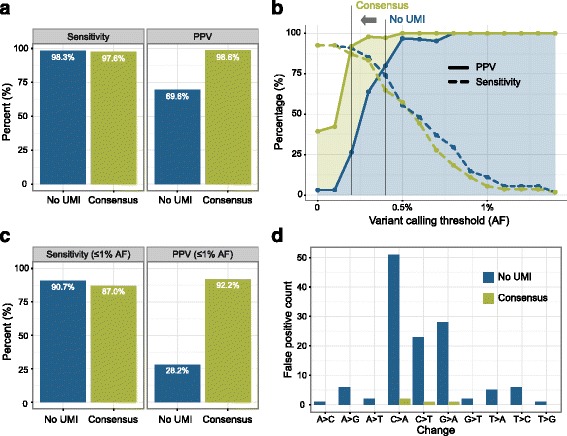
UMI consensus calling improves variant detection accuracy, allows for the detection of rare variants, and corrects 8-oxoguanine errors. **a** Sensitivity and positive predictive value (PPV) using either no UMI or consensus calling for expected allele frequencies from 0.5–99.5% across 291 SNPs. Variant calling performed using VarDict with a threshold of 0.2%. **b** Variant calling thresholds (AF) with no UMI or consensus calling further improved sensitivity and PPV for low frequency variants (*N* = 54). There were 10 and 44 sites expected at 1% and 0.5% allele frequencies, respectively. **c** Sensitivity and PPV for low frequency variants expected at AF 0.5–1% when using no UMI versus consensus calling using a variant calling threshold of 0.2% with VarDict. **d** Number of false positive calls with or without UMI consensus using a variant calling threshold of 0.2%

To test the application of the UMIs in the dual-matched adapters, a library prepared from a 1% mixture of the genomically well-characterized NA12878/NA24385 samples (25 ng, 0.5% minimum alternate allele) from the National Institute of General Medical Sciences (NIGMS) Human Genetic Cell Repository was used to model low-frequency variants and was enriched using a 75 kb custom xGen IDT Lockdown Panel. This library was sequenced on an Illumina MiSeq v2 flow cell to a mean target coverage of 1500× (Picard deduplicated) using 18 million reads. Consensus analysis, whereby reads sharing the same genomic coordinates and UMI sequence were collapsed to build consensus reads, improved variant detection (Fig. 6a). Sensitivity and positive predictive value (PPV) were assessed across the Genome in a Bottle (GIAB, http://jimb.stanford.edu/giab/), high-confidence region (35 kb) with a variant calling threshold of 0.2%. Consensus analysis improved the sensitivity and PPV across a range of variant calling thresholds (Fig. 6b), and using a minimum of three reads to build a consensus greatly improved PPV for variants present at 0.5–1% allele fraction (Fig. 6c). Base substitutions derived from 8-oxoguanine (C > A/G > T transversions) artifacts, which are common in formalin-fixed paraffin embedded (FFPE) samples, were corrected using consensus analysis (Fig. 6d) [19]. UMI consensus analysis combined with mutation-specific thresholds (Additional file 10: Figure S10a) for oxidative damage improved the PPV for low-frequency (<1%) variants from 28% to 96% while retaining a sensitivity of 87% for true positives (Additional file 10: Figure S10b).

## Discussion

There is an increasing need for sensitive and accurate detection of subclonal populations in MPS research projects and clinical assays, such as in ctDNA variant detection and microbial infection in human samples. Samples commonly undergo library construction using an index for each sample followed by pooling for capture and/or sequencing on one or more HiSeq lanes to save time and cost. Unfortunately, the likelihood of reporting false positives due to index cross-talk is a reality due to many factors, including contamination or synthesis errors during production, errors during library construction, amplification, sequencing, analysis, etc. Oligo manufacturers attempt to reduce the risk of error by creating indices based on Levenshtein distance to account for substitutions and indels, avoiding homopolymers, matching GC content, excluding self-complements, and being mindful of color balance and sequencing platform (two- versus four-color) [9]. Nevertheless, index cross-talk has been observed at levels from 0.06–10% of the sequencing reads, which greatly inhibits the end user’s ability to detect low allele fraction events and prohibits using MPS for sensitive applications.

Recently, IDT designed dual-matched indexed sequencing adapters with UMIs that are compatible with Illumina library construction and sequencers. Each individual adapter has identical i5 and i7 indices, edit distance ≥3, have 50% GC content, and are designed for both two- and four-color sequencers. Of note, there are several sample index strategies available when developing unique dual index adapters. The indices can be the same i5 and i7 sequence (i.e., dual-matched) or they could be unrelated sequences. A recent online post from Illumina suggested that unrelated sample indices are more robust than dual-matched indices [20]. However, the dual-matched index design used in our current study did not exhibit the reduced Q30 scores in the second index read that Illumina reported (data not shown). Regardless of the index pairing strategy used for sample identification, unique dual indices mitigate read misassignment in MPS assays.

When using the dual-matched indices in our experiments, cross-talk was virtually eliminated. Indeed, only one library fragment was misassigned (7.1 × 10^-7% cross-talk) when using these indices versus up to 0.3% misassigned indices when using standard, combinatorial indices (up to 49,533 or 548,358 misassigned reads to a single index on the non-patterned or patterned flow cells, respectively).

In multiplexing experiments using the dual-matched indices, we observed 0.09–0.39% contamination levels where either i5 or i7 was individually mismatched, but not both; however, these reads would be filtered out of analysis when using the matched dual-index adapters. As previously described, we observed an increase in index hopping between the HiSeq 2500 and HiSeq 3000/4000 platforms; however, the dual-matched indices would effectively mitigate the risk of incorrect sample assignment on both the patterned and non-patterned flow cells. Finally, UMI consensus analysis with the dual-matched adapters allowed for low-frequency variant detection (≥ 0.5%), and 8-oxoguanine artifacts could be corrected when using consensus analysis combined with mutation-specific thresholds.

Of course, there is risk of pre-library construction sample contamination due to sample handing errors, lab contamination, etc., and the use of dual-matched indices will not resolve this issue. For pre-library construction, cross-sample contamination, an informatics approach must be taken to detect sample concordance and contamination, such as using a publicly available analysis tool (e.g., Conpair) [21]. Additionally, the extra cost of purchasing hundreds of unique, dual-indexed adapters with UMIs relative to standard single- or dual-indexed adapters for multiplex sequencing may be unnecessary if the sensitivity provided by the unique, dual-matched adapters is not required for the experimental application.

## Conclusion

This is the first study to report the efficacy of using dual-matched indexed adapters with UMIs to virtually eliminate index cross-talk in Illumina sequencing, which is frequently observed when using standard single- or dual-indexed (combinatorial) adapter oligos. This approach will allow for the sensitive detection of low fraction sequencing events with low false positive rates, which will benefit many researchers using MPS for sensitive applications, such as microbial detection in human samples, ancient DNA sequencing, and low allele fraction somatic variant detection. This is of particular importance in microbial detection in human samples where a sample is deemed “positive” or “negative” based on only a few sequencing reads; thus, contaminating reads from nearby wells lead to false positive calls. Furthermore, using matched dual-indexed adapters with UMIs will be of great clinical benefit in cancer precision medicine for the accurate detection somatic variants in multiplexed samples and samples with low tumor purity, for the detection of low allele fraction events, and for variant calling in ctDNA.

## Methods

### Library construction, hybrid capture, sequencing

A total of 100 ng (cell lines and PDX samples) or 200 ng (FFPE samples) of extracted genomic DNA (gDNA) was used as template for library construction and prepared as previously described [22]. Briefly, gDNA was fragmented to 250 base pairs using Covaris Ultrasonication (LE220 Focused-ultrasonicator, Covaris, Woburn, MA), and fragmented DNA was purified using Agencourt AMPure XP beads (Beckman Coulter, Inc. Indianapolis, IN). Size-selected DNA was then ligated to sequencing adaptors using sample-specific indices (Illumina TruSeq HT, IDT-synthesized TS-96, or IDT dual-matched indices with UMIs), libraries were constructed using Kapa HTP (Kapa Biosystems, Wilmington, MA) and quantified using qPCR (Kapa Biosystems). The SureSelect Target Enrichment System (Agilent Technologies, Santa Clara, CA) was used to perform hybrid capture using either the SureSelect Human All Exon V5 bait set for whole exome enrichment, or smaller, custom-designed bait sets. For the multiplexing, index-hopping experiments, the libraries were prepared using the IDT dual-matched indexed adapters with the Kapa Hyper Prep kit (Kapa Biosystems), and the xGen AML Cancer Panel was used for capture (Integrated DNA Technologies, Inc., Coralville, IA). Sequencing platforms used (Illumina MiSeq, HiSeq 2500, HiSeq 3000/4000, or NextSeq) are listed along with the experiments (Illumina Inc., San Diego, CA).

### Cross-talk analysis

Each sequencing lane was demultiplexed using Picard tools ‘ExtractIlluminaBarcodes’ and ‘IlluminaBasecallsToSam’ (http://broadinstitute.github.io/picard/command-line-overview.html#Overview) [23]. To determine the amount and pattern of crosstalk, all index sequences in each sample set, including all unused indices, were used when demultiplexing. Reads that were demultiplexed with an index that was not used in library preparation were used as evidence of cross-talk. Only reads that passed the Illumina HiSeq Control Software (HiSeq 2500: HCS v.2.2.70; HiSeq 3000/4000: HCS v.3.3.52) thresholds and did not have any index base mismatches were counted. These data are presented in the same 96-well adapter plate format for each figure.

To determine the amount of underlying crosstalk in the IDT dual-matched indices, sequencing runs were demultiplexed using all possible combinations of the 35 provided indices (i.e., 35 × 35 = 1225). The number of reads demultiplexed from mismatched indices were used as evidence of cross-talk.

### Index-hopping in multiplexed captures analysis

Unique, dual-matched indexed UMI adapters were used to measure index hopping in multiplexed captures. Sample reads were demultiplexed using all possible index combinations (i.e., 16 × 16 = 256) used in an experiment. For demultiplexing, a custom Python tool was used to assign observed sequence indices to the expected list of indices, where one mismatch per index was allowed. Template hopping events were identified by counting the frequency of unexpected index pairings found (e.g., i5 index 1 + i7 index 2).

### Unique molecular indices analysis

As described above, we demultiplexed reads and created unmapped BAM files using Picard tools; UMI tags were stored to the RX tag. Reads in the unmapped BAM were aligned to the reference sequence hg19; UMI tags in the unmapped BAM file were added to the mapped BAM file using Picard MergeBamAlignment. Consensus reads were built using fgbio tools (http://fulcrumgenomics.github.io/fgbio/): GroupReadsByUmi “--strategy = adjacency --edits = 1 --min-map-q = 10”, CallMolecularConsensusReads “--error-rate-post-umi = 30 --min-reads = 1”, and FilterConsensusReads “--reverse-per-base-tags=true --min-reads=3 -E 0.05 -N 40 -e 0.1 -n 0.1.” Consensus reads were realigned and variant calling was performed with VarDict using a variant calling threshold of 0.2%. Where indicated, a mutation-specific threshold of 0.3% was applied for C > A/G > T substitutions to reduce oxoG artifacts.

## Additional files

Additional file 1: Figure S1. Proposed mechanism for index hopping. Residual-free index adapter or incomplete PCR extension products (orange) may anneal to a different template molecule (blue). Index hopping molecules will contain mismatched i5 and i7 sample indices. (PDF 818 kb)

Additional file 2: Figure S2. Standard sample indexing and library construction. (a) Plate layout of standard combinatorial indexed adapters. A total of 20 different adapters are used to create 96 unique indices, such that the i5 and i7 sequences are different in each plate well. All wells in a single column share the same i7 index. All wells in a single row share the same i5 index. Prior to library construction, i5 and i7 adapter oligonucleotides are annealed to create Y-adapters. (b) During standard library preparation, Y-adapters are ligated to sheared, end repaired, A-tailed, genomic DNA. Combinatorial indices are depicted in this image. (PDF 868 kb)

Additional file 3: Figure S3. Schematic of the sources of sample cross-talk including adapter contamination, index hopping during multiplex target enrichment post-capture PCR, index hopping during cluster amplification, and demultiplexing errors. Unique dual-matched indices reduce read misassignment because unexpected index combinations are removed from downstream analysis. (PDF 936 kb)

Additional file 4: Figure S4. Level of cross-talk using combinatorial indices on Illumina HiSeq 2500. The 96-well plate layout represents the adapter plate. A total of 19 patient-derived xenograft (PDX) libraries were prepared using IDT-synthesized TS-96 adapters (green), libraries were pooled, hybrid captured using a custom bait set, and then sequenced on a single lane of an Illumina HiSeq2500 flow cell. Numbers in each well represent the number of fragments that passed standard Illumina filters and demultiplexed using only perfect sequence matches on all TS-96 indices. (PDF 192 kb)

Additional file 5: Figure S5. Level of cross-talk using unique, dual-matched indexed adapters on Illumina HiSeq 2500. The 96-well plate layout represents the adapter plate. A total of 35 adapters were synthesized. Thirty-four samples consisting of PDX (*n* = 4), tissue samples (*n* = 20), and cancer cell lines (*n* = 10) underwent library construction using IDT-synthesized unique, dual-matched indexed adapters (green). Libraries were pooled, hybrid captured using a custom bait set, and then sequenced on a single lane of an Illumina HiSeq 2500 flow cell. Numbers in each well represent the number of fragments that passed standard Illumina filters and demultiplexed using only perfect sequence matches on all TS-96 indices. (PDF 184 kb)

Additional file 6: Figure S6. Schematic of the experiment used to measure adapter contamination and multiplexed capture index hopping. Replicate libraries were prepared using 16 unique dual-matched UMI adapters and enriched with the IDT xGen AML Cancer Panel in pools of 1, 4, 8, and 16. Each multiplexing experiment was sequenced on separate Illumina NextSeq runs. (PDF 819 kb)

Additional file 7: Figure S7. Multiplex captures have comparable uniformity to individual captures. Uniform coverage enables accurate variant calling with minimal sequencing cost. (PDF 115 kb)

Additional file 8: Figure S8. Adapter plate layout for multiplexing experiments. (PDF 829 kb)

Additional file 9: Figure S9. Comparing the level of cross-talk using combinatorial adapters between the Illumina HiSeq 2500 and Illumina HiSeq 3000/4000 platforms. The 96-well plate layout represents the adapter plate. Two samples underwent library construction using IDT-synthesized TS-96 adapters (green). Libraries were pooled, hybrid captured using a whole exome capture bait set, and then sequenced on a single lane of an (a) Illumina HiSeq2500 flow cell or (b) Illumina HiSeq 3000/4000 patterned flow cell. Numbers in each well represent the number of fragments that passed standard Illumina filters and demultiplexed using only perfect sequence matches on all IDT TS-96 indices. (PDF 210 kb)

Additional file 10: Figure S10. Mutation-specific thresholds provide additional improvements to calling accuracy. (a) Number of false positives from 8-oxoguanine errors are found at low frequencies. (b) Increased minimum variant allele frequency thresholds for 8-oxoguanine mutations improves the positive predictive value (PPV) for rare variants without reducing sensitivity. (PDF 168 kb)

## Abbreviations

ctDNA: circulating cell-free tumor DNA
FFPE: Formalin-fixed paraffin embedded
gDNA: genomic DNA
GIAB: Genome in a Bottle
IDT: Integrated DNA Technologies, Inc.
MPS: Massively parallel sequencing
NIGMS: National Institute of General Medical Sciences
PDX: Patient-derived xenografts
UMI: Unique Molecular Indices
