# Supplementary figures and images for: Unique, dual-indexed sequencing adapters with UMIs effectively eliminate index cross-talk and significantly improve sensitivity of massively parallel sequencing
Source: BMC Genomics. 2018 Jan 8;19:30. doi: 10.1186/s12864-017-4428-5 (PMC5759201; doi:10.1186/s12864-017-4428-5)

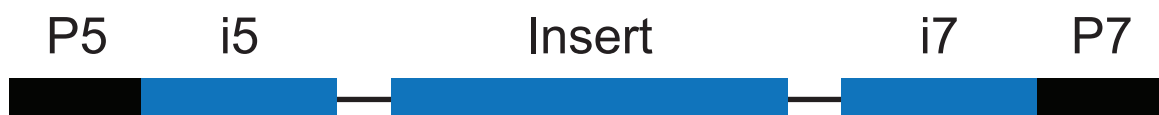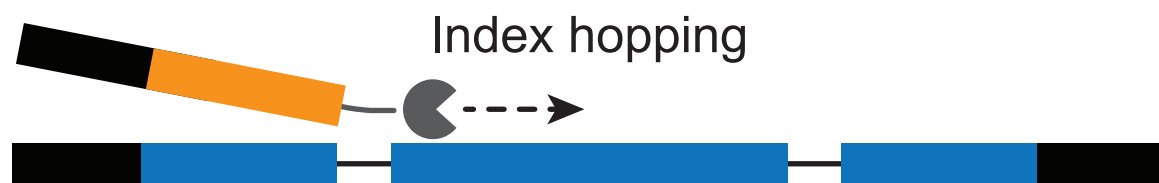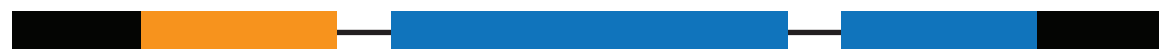

Supplement: Supplementary file 1 — Proposed mechanism for index hopping. Residual-free index adapter or incomplete PCR extension products (orange) may anneal to a different template molecule (blue). Index hopping molecules will contain mismatched i5 and i7 sample indices. (PDF 818 kb) [file 12864_2017_4428_MOESM1_ESM.pdf]

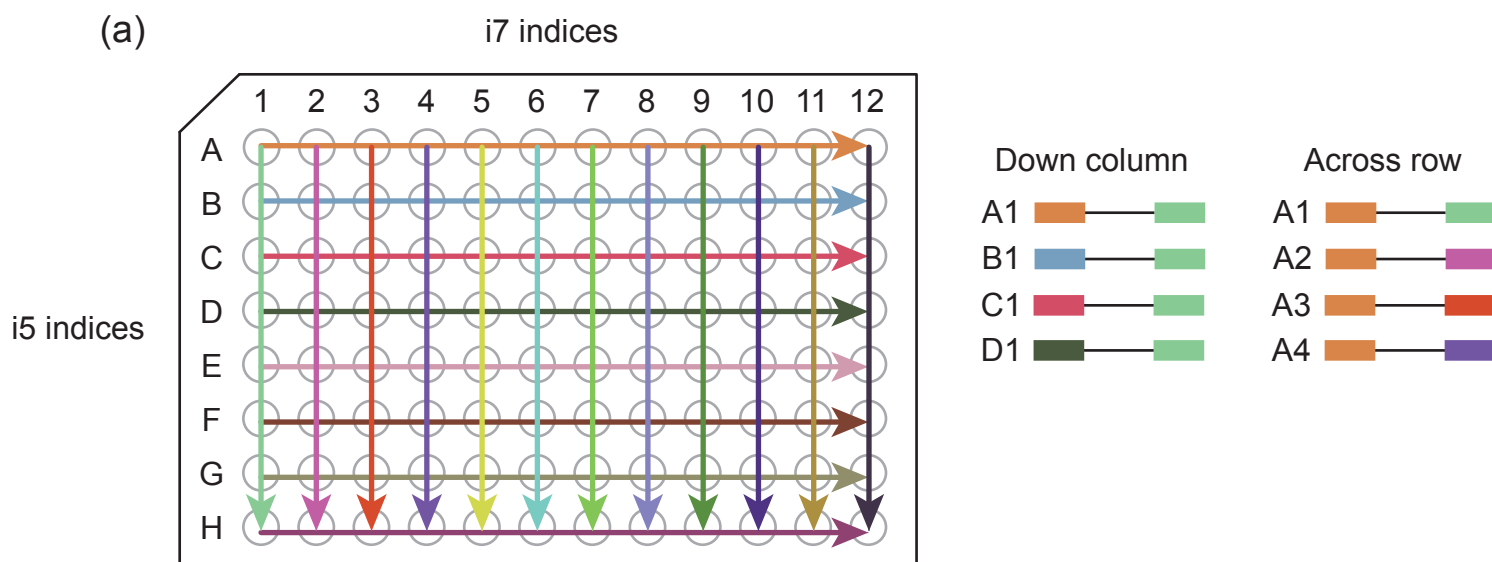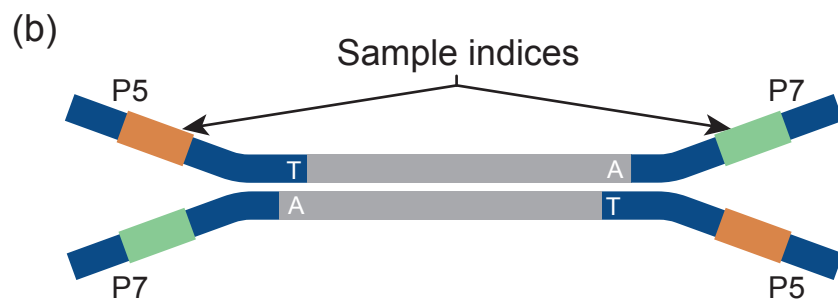

Supplement: Supplementary file 2 — Standard sample indexing and library construction. (a) Plate layout of standard combinatorial indexed adapters. A total of 20 different adapters are used to create 96 unique indices, such that the i5 and i7 sequences are different in each plate well. All wells in a single column share the same i7 index. All wells in a single row share the same i5 index. Prior to library construction, i5 and i7 adapter oligonucleotides are annealed to create Y-adapters. (b) During standard library preparation, Y-adapters are ligated to sheared, end repaired, A-tailed, genomic DNA. Combinatorial indices are depicted in this image. (PDF 868 kb) [file 12864_2017_4428_MOESM2_ESM.pdf]

16 libraries

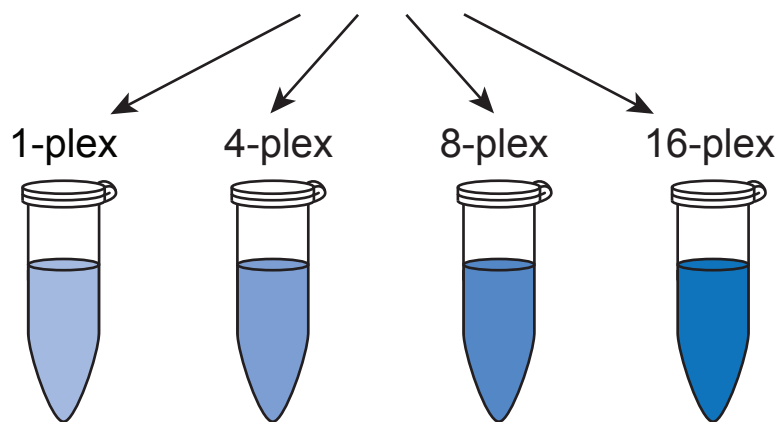

Supplement: Supplementary file 6 — Schematic of the experiment used to measure adapter contamination and multiplexed capture index hopping. Replicate libraries were prepared using 16 unique dual-matched UMI adapters and enriched with the IDT xGen AML Cancer Panel in pools of 1, 4, 8, and 16. Each multiplexing experiment was sequenced on separate Illumina NextSeq runs. (PDF 819 kb) [file 12864_2017_4428_MOESM6_ESM.pdf]

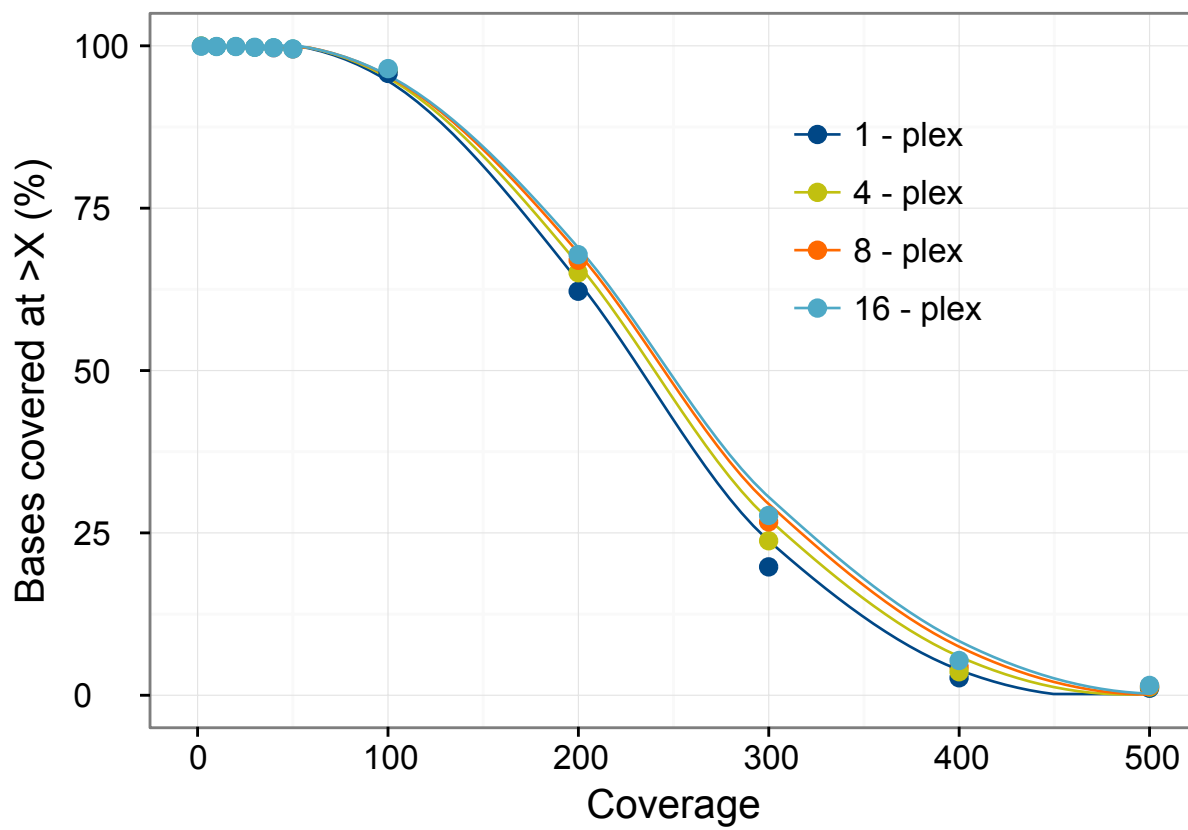

Supplement: Supplementary file 7 — Multiplex captures have comparable uniformity to individual captures. Uniform coverage enables accurate variant calling with minimal sequencing cost. (PDF 115 kb) [file 12864_2017_4428_MOESM7_ESM.pdf]

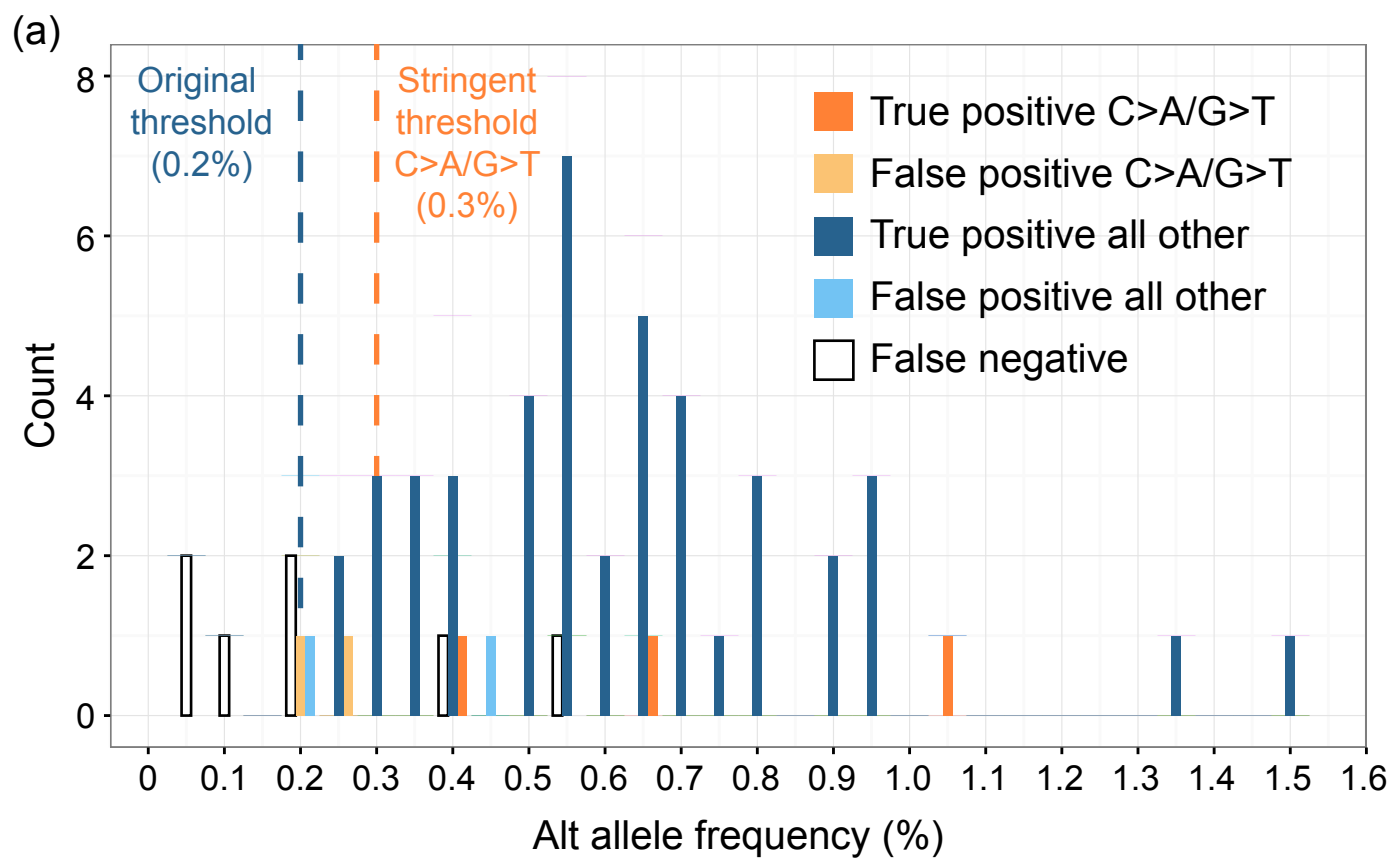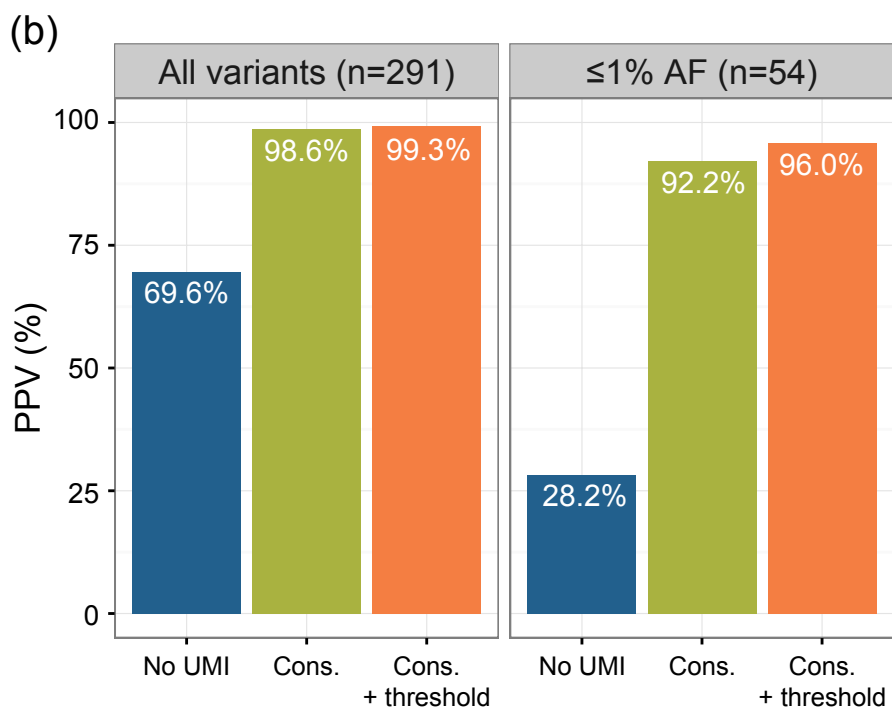

Supplement: Supplementary file 10 — Mutation-specific thresholds provide additional improvements to calling accuracy. (a) Number of false positives from 8-oxoguanine errors are found at low frequencies. (b) Increased minimum variant allele frequency thresholds for 8-oxoguanine mutations improves the positive predictive value (PPV) for rare variants without reducing sensitivity. (PDF 168 kb) [file 12864_2017_4428_MOESM10_ESM.pdf]
