# Supplementary material for: Unique, dual-indexed sequencing adapters with UMIs effectively eliminate index cross-talk and significantly improve sensitivity of massively parallel sequencing
Source: BMC Genomics. 2018 Jan 8;19:30. doi: 10.1186/s12864-017-4428-5 (PMC5759201; doi:10.1186/s12864-017-4428-5)
Supplement: Supplementary file 3 — Schematic of the sources of sample cross-talk including adapter contamination, index hopping during multiplex target enrichment post-capture PCR, index hopping during cluster amplification, and demultiplexing errors. Unique dual-matched indices reduce read misassignment because unexpected index combinations are removed from downstream analysis. (PDF 936 kb) [file 12864_2017_4428_MOESM3_ESM.pdf]

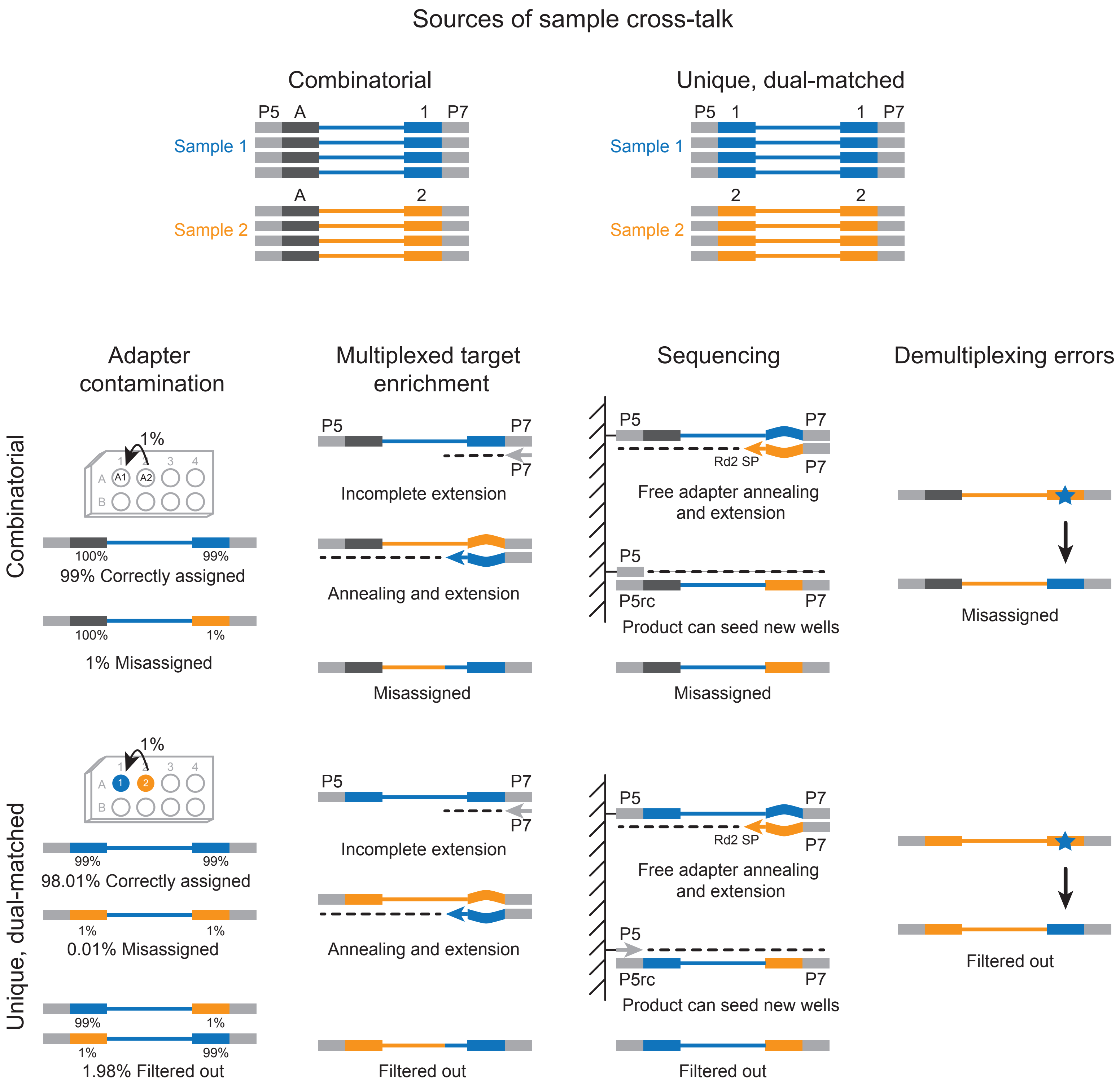
Sources of sample cross-talk
Combinatorial
Unique, dual-matched
P5
A
1
P7
Sample 1
2
Sample 2
Adapter contamination
Multiplexed target enrichment
Sequencing
Demultiplexing errors
Combinatorial
1%
A1
A2
3
4
B
100%
99%
99% Correctly assigned
1%
1% Misassigned
Incomplete extension
Annealing and extension
Misassigned
Rd2 SP
Free adapter annealing and extension
P5rc
Product can seed new wells
Unique, dual-matched
98.01% Correctly assigned
0.01% Misassigned
1.98% Filtered out
Filtered out
