# Supplementary material for: Unique, dual-indexed sequencing adapters with UMIs effectively eliminate index cross-talk and significantly improve sensitivity of massively parallel sequencing
Source: BMC Genomics. 2018 Jan 8;19:30. doi: 10.1186/s12864-017-4428-5 (PMC5759201; doi:10.1186/s12864-017-4428-5)
Supplement: Supplementary file 4 — Level of cross-talk using combinatorial indices on Illumina HiSeq 2500. The 96-well plate layout represents the adapter plate. A total of 19 patient-derived xenograft (PDX) libraries were prepared using IDT-synthesized TS-96 adapters (green), libraries were pooled, hybrid captured using a custom bait set, and then sequenced on a single lane of an Illumina HiSeq2500 flow cell. Numbers in each well represent the number of fragments that passed standard Illumina filters and demultiplexed using only perfect sequence matches on all TS-96 indices. (PDF 192 kb) [file 12864_2017_4428_MOESM4_ESM.pdf]

Colour	Meaning
Green	Barcode used in sample library preparation
Yellow	Row / column cross-talk
Red	Non row / column cross-talk
White	No cross-talk

	1	2	3	4	5	6	7	8	9	10	11	12
**A**	19314405	10758720	7505334	774	221	1779	107	15	31	1114	74	7
**B**	7083259	6992761	7272206	537	108	746	45	17	1562	1036	41	175
**C**	7496941	7749888	10852110	864	76	558	57	3	25	1410	32	163
**D**	7045812	7850625	4922	23	76	476	46	18	26	191	27	125
**E**	7204634	7364015	3177	87	394	544	198	134	454	440	687	205
**F**	7602197	6660412	2999	22	31	579	25	6	28	161	25	0
**G**	8133248	7805134	3456	13	62	588	80	9	17	181	35	0
**H**	6830509	8737064	3163	6	64	446	79	5	24	207	38	0
