# Supplementary material for: Unique, dual-indexed sequencing adapters with UMIs effectively eliminate index cross-talk and significantly improve sensitivity of massively parallel sequencing
Source: BMC Genomics. 2018 Jan 8;19:30. doi: 10.1186/s12864-017-4428-5 (PMC5759201; doi:10.1186/s12864-017-4428-5)
Supplement: Supplementary file 8 — Adapter plate layout for multiplexing experiments. (PDF 829 kb) [file 12864_2017_4428_MOESM8_ESM.pdf]

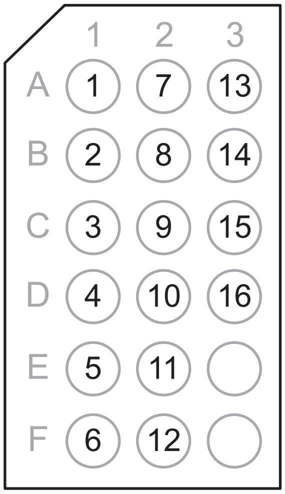
1 2 3
A 1 7 13
B 2 8 14
C 3 9 15
D 4 10 16
E 5 11
F 6 12
