# Supplementary material for: Unique, dual-indexed sequencing adapters with UMIs effectively eliminate index cross-talk and significantly improve sensitivity of massively parallel sequencing
Source: BMC Genomics. 2018 Jan 8;19:30. doi: 10.1186/s12864-017-4428-5 (PMC5759201; doi:10.1186/s12864-017-4428-5)
Supplement: Supplementary file 9 — Comparing the level of cross-talk using combinatorial adapters between the Illumina HiSeq 2500 and Illumina HiSeq 3000/4000 platforms. The 96-well plate layout represents the adapter plate. Two samples underwent library construction using IDT-synthesized TS-96 adapters (green). Libraries were pooled, hybrid captured using a whole exome capture bait set, and then sequenced on a single lane of an (a) Illumina HiSeq2500 flow cell or (b) Illumina HiSeq 3000/4000 patterned flow cell. Numbers in each well represent the number of fragments that passed standard Illumina filters and demultiplexed using only perfect sequence matches on all IDT TS-96 indices. (PDF 210 kb) [file 12864_2017_4428_MOESM9_ESM.pdf]

(green)	Barcode used in sample library preparation
(yellow)	Row / column cross-talk
(red)	Non row / column cross-talk
(white)	No cross-talk

(a)

	1	2	3	4	5	6	7	8	9	10	11	12
**A**	41519	54198466	6	7	0	8	491	4	150	1057	11	1
**B**	54107457	49533	221	1	500	4479	115	10	29	125	9	0
**C**	8	11	7	1	0	0	0	0	0	0	0	0
**D**	11027	16	0	0	0	0	0	0	0	0	0	0
**E**	7	557	1	0	1	0	1	0	0	0	1	0
**F**	5	12	1	0	0	0	0	0	0	0	0	0
**G**	3	214	0	0	1	1	0	0	1	0	0	0
**H**	10	18	0	0	0	0	0	0	0	0	0	0

(b)

	1	2	3	4	5	6	7	8	9	10	11	12
**A**	548358	197176658	10	2	8	44	1867	1	554	4872	5	2
**B**	184166643	506396	852	0	1469	14560	364	26	64	465	0	1
**C**	15	125	1	2	0	0	0	0	0	1	0	2
**D**	38047	133	0	1	0	4	0	0	0	6	0	0
**E**	20	1960	3	0	3	2	3	0	0	0	9	0
**F**	10	12	0	0	0	0	0	0	1	0	0	0
**G**	6	746	1	0	0	0	0	0	1	0	0	0
**H**	16	21	2	0	0	0	1	0	0	0	0	0
