# Supplementary material for: Unique, dual-indexed sequencing adapters with UMIs effectively eliminate index cross-talk and significantly improve sensitivity of massively parallel sequencing
Source: BMC Genomics. 2018 Jan 8;19:30. doi: 10.1186/s12864-017-4428-5 (PMC5759201; doi:10.1186/s12864-017-4428-5)
Supplement: Supplementary file 5 — Level of cross-talk using unique, dual-matched indexed adapters on Illumina HiSeq 2500. The 96-well plate layout represents the adapter plate. A total of 35 adapters were synthesized. Thirty-four samples consisting of PDX (n = 4), tissue samples (n = 20), and cancer cell lines (n = 10) underwent library construction using IDT-synthesized unique, dual-matched indexed adapters (green). Libraries were pooled, hybrid captured using a custom bait set, and then sequenced on a single lane of an Illumina HiSeq 2500 flow cell. Numbers in each well represent the number of fragments that passed standard Illumina filters and demultiplexed using only perfect sequence matches on all TS-96 indices. (PDF 184 kb) [file 12864_2017_4428_MOESM5_ESM.pdf]

(green)	Barcode used in sample library preparation
(yellow)	Row / column cross-talk
(red)	Non row / column cross-talk
(white)	No cross-talk
(grey)	Adapter not synthesized

	1	2	3	4	5	6	7	8	9	10	11	12
**A**	4086039	4213526	4073731	3673722	3342951	3716997	3454644	3013793	3526714	3703973	3630011	3401172
**B**	2545444	2093187	2959143	9629730	3410286	3424278	3466314	3401125	3680707	3123801	2902095	3470507
**C**	3203521	3688996	3174886	3284897	3289110	2985313	3278669	3300028	3247516	3467130	0	
**D**												
**E**												
**F**												
**G**												
**H**												
